# Financial value regime alignment and Mozambique’s heterogeneous energy landscape

**DOI:** 10.1007/s11625-025-01687-7

**Published:** 2025-05-11

**Authors:** Idalina Baptista, Lorraine Howe, Carlos Shenga

**Affiliations:** 1 Department for Continuing Education, University of Oxford, Rewley House, 1 Wellington Square, Oxford, OX1 2JA UK; 2 Centre for Research on Governance and Development, Maputo, Mozambique

**Keywords:** Energy transition, Renewable energy, Heterogeneous infrastructures, Financialization, Creditworthiness, Bankability

## Abstract

Meeting the UN’s Sustainable Development Goal 7 of universal access to affordable, reliable, sustainable and modern energy for all by 2030 will require an unprecedented amount of private sector finance and development assistance. To this end, African utilities and governments are asked to put substantive efforts into aligning themselves with the demands of the dominant financial value regime of ‘the Wall Street Consensus’ (Gabor in Dev Change 52:429–459, 2021). Using the case of Mozambique, this article examines the strategies deployed by state actors to align themselves with this regime and the implications this has for the country’s energy landscape, particularly in terms of environmental sustainability and social justice. It examines how two of the regime’s principles—creditworthiness and bankability—permeate new legislative efforts in the on-grid and off-grid sectors, organizational structures, accounting and project management practices, attempts at disciplining workers and consumers, and the broader political economy of Mozambique. It also discusses how such efforts have perpetuated a fragmented energy landscape that does not always satisfy the needs of energy poor populations or facilitate decarbonization. The article concludes with a discussion of the relevance of our findings to crafting environmentally sustainable and socially just pathways for Africa’s electrification. The article draws on the authors’ ongoing field research in Mozambique, including the qualitative analysis of existing policies and of eight in-depth, semi-structured interviews with key informants (energy operators, donors and implementing partners) undertaken in late 2022.

## Introduction

Africa’s odds of meeting the UN’s Sustainable Development Goal 7 (SDG7)—of universal access to affordable, reliable, sustainable and modern energy for all by 2030—seem rather steep now. According to the Afrobarometer (2024), only 44% of Africans have access to a reliable electricity connection, varying significantly between urban (65%) and rural (24%) areas. Despite substantive improvements over the last two decades, an estimated annual investment of USD 64 billion is needed (AfDB 2024). National utilities and governments cannot meet this challenge through their budgets alone. They require access to an unprecedent amount of private sector finance (either through debt or equity) and bilateral and multilateral development assistance (e.g., concessional loans, grants or guarantees by multinational development banks such as the World Bank or via bilateral aid from individual countries).

To this end, the prevailing consensus among development and climate policymakers is that substantive efforts must go into mitigating the perceived risks of investing in African energy infrastructure (IRENA and AfDB 2022; The World Bank 2017). In particular, African utilities and governments must put substantive efforts in ‘de-risking’ their political environments (i.e., to offer a sense of political stability to minimize the potential for financial losses), making themselves more creditworthy (i.e., capable of borrowing from the private sector), as well as preparing a pipeline of bankable projects (i.e., projects ready to be funded) (AfDB 2016; White and Wahba 2019). This ‘Wall Street Consensus’, as Gabor (2021) puts it, is not exclusive to the electricity sector. It is now a dominant financial value regime shaping the financialization of infrastructures and of development more broadly (Chiapello et al. 2023; Mawdsley 2018).

The efforts of aligning Africa’s electricity sector with this dominant financial value regime come with challenges over its environmental sustainability and socio-spatial equity. On the one hand, infrastructures have become an appealing global asset class (Weber et al. 2016), whereby the investment is largely disconnected from the environmental, social or economic purposes of infrastructures on the ground (Bear 2020). Even when investors see themselves as playing a key role in promoting sustainability or climate mitigation/adaptation (Langley 2020), interests other than those of local or vulnerable communities often take precedence (Galindo-Gutiérrez 2024; Venner et al. 2024). On the other hand, the financialization of infrastructures has strong socio-spatial dimensions, particularly in terms of what infrastructure gets build, where, how, and for whom (Castree and Christophers 2015). Africa is no stranger to the spatial unevenness of infrastructure networks that endures since colonial times (van der Straeten and Hasenöhrl 2016). In the absence of formal electricity networks, many Africans satisfy their energy needs through a heterogeneous combination of fuels and modes of supply of varying degrees of formality and probity (Jaglin 2014; Lawhon et al. 2018). Rural populations and/or lower income groups who are either too distant from existing grids or have limited ability to pay for existing services are particularly affected. Despite growing efforts to bridge this gap through off-grid/renewables solutions, finance for these less profitable market segments is yet to reach its apparent potential (IRENA 2021). In this context, national utilities and governments must constantly negotiate their alignment with the demands of potential investors alongside citizens’ demands for better and affordable electricity services and global calls for decarbonization of the electricity sector (Alami et al. 2023).

Using the case of Mozambique, this article examines the strategies deployed by state actors to align themselves with the dominant financial value regime and the implications this has for the country’s energy landscape, particularly in terms of environmental sustainability and social justice. As we demonstrate in “Results and discussion: value alignments, complex entanglements”, Mozambique offers an excellent opportunity to examine the processes through which state actors negotiate this alignment and its effects. Drawing on a qualitative analysis of existing policies and key informant semi-structured interviews, the article examines the efforts of the Government of Mozambique (GoM) and the state-owned national electricity utility EDM (*Electricidade de Moçambique, E.P.*) to improve the attractiveness of on-grid and off-grid sectors. Specifically, it examines how utility creditworthiness and project bankability permeate not just the organizational structure of EDM but also accounting and project management practices, attempts at disciplining workers and consumers, and new legislative efforts to create competitive electricity markets. The article discusses how such efforts have perpetuated a fragmented energy landscape that does not always satisfy the needs of energy poor populations or facilitate decarbonization.

The article begins by foregrounding ongoing debates about the financialization of Africa’s infrastructure development within broader challenges regarding the heterogeneity of infrastructure services across the continent, how to govern it, and with what effects. It then provides an overview of the methodology, before proceeding with the analysis of the processes of alignment with the dominant financial value regime in the on-grid and off-grid sectors and its effects. The article concludes with a discussion of the relevance of our findings to crafting environmentally sustainable and socially just pathways for Africa’s electrification.

## Financial value regimes and heterogeneous energy infrastructures

It is now widely accepted that most African countries experience varying degrees of heterogeneity in the supply of and access to basic services, such as electricity, water, or transport (Lawhon et al. 2018). African populations satisfy their needs through a diversity of service configurations where formal (public or private) providers are but one of the options available (Jaglin 2014). These include a combination of fuels (e.g., charcoal, electricity, gas), of sources (e.g., on-grid electricity, personal generator, home solar system), and of diverse levels of regulatory probity (e.g., paying to a regulated formal supplier, illegal connections to the grid, or paying a neighbor to access their formal on-grid connection) (Munro et al. 2020). Diverse service configurations tend to prevail in contexts of uncertainty and limited resources, in which the state is unable (or unwilling) to provide for formal services, or to adequately regulate private provision, or where services available are unavailable or unaffordable, especially to rural and/or lower income users.

Such heterogeneity is neither inherently negative nor positive but signals the variety of challenges that utilities and governments face in ensuring access to modern energy services in the context of scarce resources. As Lawhon et al. (2023) suggest, some governments are under pressure from citizens to come to terms with such heterogeneous service configurations, which they once perceived as undesirable, informal or illegal. Jaglin (2008) has long suggested that service differentiation may be a pragmatic and potentially progressive choice to respond to the diverse needs of the urban poor that the Euro-American notions of ‘universal service’ or ‘welfare state’ may be ill-equipped to address. Her intervention relates to broader efforts to bring postcolonial and de-colonial thinking to bear on the multiple meanings of energy justice, especially in post-colonial contexts (Castán Broto et al. 2018; Tornel 2023). Despite its potential flexibility, heterogeneity of service provision can be complex to govern and offers no guarantees of curbing existing inequality and environmental degradation among more vulnerable populations (Lawhon et al. 2023). Whether the negative impacts of heterogeneity prevail across the continent will depend largely on the ability of African governments to close the infrastructure gap in ways that improve the affordability and quality of electricity services across their territories. As noted earlier, meeting SDG7 demands a capital investment that African governments cannot fulfill without the support of private finance (debt or equity) and bilateral or multilateral development assistance (AfDB 2024).

There is now an abundance of policy efforts to attract such investment, especially in support of the energy transition from high-carbon to renewable energy (IRENA and AfDB 2022). These efforts have been in the making since the 1980s, when the World Bank began supporting electricity sector reforms across Africa (The World Bank 1993). The reforms range from the longstanding efforts to create competitive electricity markets, enhance countries’ business environments, and improve the governance and creditworthiness of state-owned utilities (Eberhard and Shkaratan 2012); to new governance models for ownership and control, such as public–private partnerships (PPPs) (Klein 2015); to providing technical assistance in preparing project pipelines and increasing project bankability (Leigland and Roberts 2007); to a range of financial instruments of increasing complexity (IRENA 2016).

While it is outside the scope of this article to discuss the arguably uneven results of these efforts, it is important to examine how they have shaped in-country institutions and the possibilities for a just sustainable energy transition. We frame this analysis in the context of extensive evidence of the growing financialization of development (Chiapello et al. 2023; Gabor 2021; Mawdsley 2018) and of the financialization of infrastructures in particular (Cirolia 2020; McArthur 2024; O’Neill 2019). We acknowledge that finance, i.e., capital supplied on the basis of profit on return, has been a mainstay of electricity infrastructure development since the nineteenth century (Hausman et al. 2008), although the amounts of finance required nowadays may be quantitatively larger. We use ‘financialization’ here to mean “the increasing dominance of financial actors, markets, practices, measurements, and narratives, at various scales, resulting in a structural transformation of economies, firms (including financial institutions), states and households” (Aalbers 2019, p. 3).

In particular, we see financialization as being operationalized through a financial value regime of semi-coherent “sets of implicit or explicit principles, norms, rules, and decision-making procedures” (Krasner 1982, p. 186). Return on investment, the minimization of risk, and portfolio diversification are some of the key principles financial actors draw on to make decisions on whether and where to invest (Weber et al. 2016). They assess the predictability and stability of recouping their investment through calculative practices such as evidence-based quantification, metrification, commensurability, and auditing (Miller 2004). Some investors may be more willing to take on higher risk for an expectation of higher return, while others may be more concerned with the environmental and social impact of their investments than those investors focused solely on profit extraction (Langley 2020). At any rate, most investors are often too far removed from the specificities of the projects they invest in. Reviewing the utility-scale renewable electricity projects in South Africa and Mexico, Baker (2022) concluded that the financial principles embedded in renewable electricity markets are rarely attentive to the contextual challenges they pose to project implementation or their meeting of social responsibility goals (see also Baker 2015). As a result, investors see infrastructures not as public goods serving public needs, but as another financial asset in their wider portfolio of investments (Weber et al. 2016).

In this context, we find useful Gabor (2021)’s labeling of the dominant financial value regime in development circles as the ‘Wall Street Consensus’. It signals the ways in which a set of powerful and self-interested financial actors and their values, calculative practices, and narratives shape development policies, practices, and agendas on a global scale (Christophers 2023). This financial value regime is performed and reproduced by the recipients of finance themselves and a host of ancillary actors, such as credit rating agencies, brokers and fund managers, accounting firms, and consultancies, whose interests may not always align (Elder-Vass 2022). Indeed, donors and multilateral development institutions, such as the World Bank or the African Development Bank, have compelled the adoption of this financial value regime through the policy reforms noted above. The reforms are often presented as conditionalities to funding energy infrastructure initiatives, including more recent off-grid/renewables investments, although it remains unclear how well they meet stated sustainability and equity aims (Baptista and Plananska 2017).

The resulting alignment with the dominant financial value regime is what some authors designate as international financial subordination (Alami et al. 2023). This can have substantive impacts on local actors’ ability to pursue alternative development pathways for sustainable energy transitions, as it impacts what infrastructure gets built, where, and for whom. However, research shows that local actors are not without agency. They seldom accede to full subordination and instead make strategic use of available mechanisms to limit, contest or subvert undesired effects of the dominant financial value regime. State actors often negotiate electricity sector reforms and alignment with financing conditionalities alongside their own economic accumulation strategies, demands from citizens, and the material reality of existing infrastructure (Alami et al. 2023; Cirolia 2020). These negotiations also have a territorial expression and extend to the material fabric of social life, particularly in urban spaces (O’Brien et al. 2019; O’Neill 2019). Irrespective of which interests prevail in such negotiations, they will have effects on a country’s energy landscape at various scales. This makes a spatial analysis of the effects of financial logics the more relevant in studies of finance in the global South.

One way of examining these power dynamics and its various (including spatial) effects is to explore the efforts of local actors to meet two key aspects of infrastructure development: creditworthiness and project bankability. Creditworthiness refers to the ability of the project owner—usually a utility, government, business consortium, or PPP—to repay its debt obligations (C40 2016; Weber et al. 2016). This is usually assessed by third parties like credit-rating agencies but relies on the demonstration of sound management and financial practices (e.g., organized accounts or annual financial audits by respected third party consultancies). Project bankability refers to the assessment that financiers make of a project’s risk-return profile and of the stability and predictability of the country’s regulatory and political environment to meet expected returns in a timely fashion (CCFLA 2022; Weber et al. 2016). Financiers deem a project bankable when it meets certain expectations, including: its business case offers evidence of a predictable and reliable revenue stream (e.g., evidence of demand from an off-taker or evidence of effective service fee collection rates); the project is professionally packaged in technically competent and financially literate language, including every aspect of its planning, development, construction and operation, environmental assessment or required permits; or the project is grounded in a rules-based institutional and regulatory environment (e.g., existence of sector-specific regulatory agencies and legislation or mechanisms to enforce contracts and property-rights) (Leigland and Roberts 2007).

Meeting these expectations has been challenging for many utilities, despite the ongoing sector reforms. Indeed, African utilities, governments, and business continue to struggle to demonstrate their creditworthiness, leading some to suggest that development banks should leverage their expertise to provide technical assistance or to provide blended finance (Simone and Bazilian 2019). Moreover, project preparation in African contexts is on average double the cost than that of developed countries’ contexts, about 10% of a project’s total investment cost (Leigland and Roberts 2007). This put African project owners at a disadvantage, since it adds to already substantive finance requirements. The challenges are even greater for green, sustainable, climate resilient infrastructure in cities (White and Wahba 2019). Therefore, understanding how African utilities and governments balance all these demands is key to illuminate the challenges toward sustainable, universal electrification across Africa. To further explore these processes and their effects, we now turn to examine the case of Mozambique.

## Methodology

This article draws on two sets of data. The first dataset concerns a compilation of official documents, legislation, policy reports, news articles, and other media pieces relating to Mozambique’s electricity sector since post-independence. The authors compiled this data set over the last decade through desk-based review of materials available online and complemented by archival research, fieldwork observations, and stakeholder semi-structured interviews undertaken over that period. While this article draws specifically on a sub-set of recent policy documents, their analysis is informed by the knowledge amassed to date. The second dataset concerns eight in-depth, semi-structured interviews with energy operators, including bilateral and multi-lateral donors and implementing partners working in Mozambique’s off-grid energy sector. These interviews were conducted in August and September 2022. The guide was designed to capture data on the opportunities and challenges presented by the new off-grid energy regulation (República de Moçambique 2021). Both data sets were analyzed using a qualitative approach to systematize key points and derive latent meanings, with feedback from key informants sought to validate the analysis of the latter interviews. The insights developed from the data analysis were compared with published work of other authors working in, and on, Mozambique, complemented with a review of government policies and gray literature from relevant institutions in the energy sector, and considered in the context of the knowledge accumulated over the years by the three authors about the country’s electricity sector.

## Results and discussion: value alignments, complex entanglements

Mozambique’s efforts toward meeting SDG7 are a good exemplar of efforts to align with the dominant financial regime at play across the continent. For the last 3 decades, Mozambique has made substantive efforts to expand access to electricity across its territory, raising it from 5% in the 1990s to 43% in 2022 (EDM 2023; Kirshner et al. 2020). However, access varied immensely across the country, with a 98% connection rate in the capital Maputo City and Province in the urbanized south, against 22% in rural Zambézia in the center (EDM 2023). With 61% of the population still living in rural areas, Mozambicans satisfy their energy needs through a diversity of service configurations, relying on a mix of fuels (firewood, charcoal, gas, electricity), engaging multiple providers (formal and informal), with different levels of quality and affordability, varying across rural/urban contexts and along income levels (Castán Broto et al. 2018; Shenga et al. 2024; Smith et al. 2022). The result is a heterogeneous energy landscape, both in terms of diversity of energy services and socio-spatial variation.

Tackling the unevenness of Mozambique’s heterogeneous energy landscape will be costly—and challenging. The GoM’s 2018 Integrated Electricity Master Plan estimated it would require an investment of USD 34 billion over 25 years, or USD 1.36 billion a year until 2043, to meet universal electrification and respond to growing demand (República de Moçambique 2018a). This means an annual investment of approximately 7% of the country’s GDP, an impossible commitment for one of the poorest countries in the world (in GDP per capita), with a tax revenue of c. 27% of its GDP and a debt-to-GDP ratio of 124% (The World Bank 2022). Mozambique’s electrification has been heavily dependent on development assistance, given the underdevelopment of its internal capital market and financial services, which are unable to provide substantive private equity or debt. For instance, the market capitalization of Mozambique’s Stock Exchange in 2021 amounted to just under USD 2 billion (Diário Económico 2022). Attracting foreign capital is also thwarted by Mozambique’s ranking as one of the least easy countries to do business in (138th out of 190 economies, according to The World Bank 2020). Similarly, domestic private energy operators in the renewables sector have limited capacity to meet the eligibility requirements to access private finance and or to absorb investment, including in terms of technical capacity and financial literacy skills (Howe et al. 2024).

This situation has deep historical roots in the colonial extractive economic model based on forced plantation labor and service provision along a few transport corridors (Kirshner and Baptista 2023; Power and Kirshner 2019). When Mozambique became independent in 1975, the country had a very fragmented electric grid, mostly limited to urban centers with an access rate of about 5%, mostly by white settlers (Dava and Tamele 2011). The four main power plants at the time—the hydropower stations of Cahora Bassa, Chicamba and Mavuzi, and the SONEFE thermal power station—had all been built through combinations of public and private investments (Dava and Tamele 2011). Until the early 1990s, Mozambique experienced substantive political, economic, and social turmoil as the newly independent leadership led the country through Socialism and a civil war (Hall and Young 1997). During this period the state managed to expand the transmission grid in the Center-North region through loans, grants or donations from countries of the Soviet Union and a few European nations, but few other investments were possible given the economic crisis that followed shortly after independence. This led the GoM to abandon Socialism in the mid-1980s and transition to a multi-party democracy in 1990, while implementing a structural adjustment program supported by the IMF that ushered in an era of neoliberal policies (Pitcher 2002). This extended to the electricity sector, which underwent substantive reforms, under the auspices of the World Bank and the donor community, with a view to attracting private funding (Shenga et al. 2024). Therefore, Mozambique has been exposed to the dominant financial value regime for several decades now. The discovery of substantive natural gas reserves in the early 2010s somewhat changed Mozambique’s fortunes, as the government sought to borrow against future gas income (Foster et al. 2024). It also threatens the country’s transition to a sustainable energy future.

Mozambique has seen several investments in the energy sector with private participation since the 1990s. Detailed investment data is piecemeal, but existing World Bank statistics offer an approximate picture: between 2003–2022, investment in energy projects with private participation for electricity and natural gas (generation, transmission, and distribution), amounted to c. USD 3 billion (The World Bank 2022). Among these projects are four fully private Independent Power Producers (IPPs) (i.e., Aggreko, Gigawatt, Kuvaninga Energia, Cuamba Solar) and three IPPs in which the national utility EDM is one of the shareholders (i.e., CTRG, Temane, Mocuba). Private partners range from energy companies accustomed to working in southern Africa (e.g., Sasol, EDF) to Mozambican holding companies and international development financiers (mainly from European countries). Alongside equity (usually as minority contributions), financing for such projects has been amassed mainly from a variety of development assistance grants, loans, and guarantees, namely from the US International Development Finance Corporation (IFC), the Emerging Africa Infrastructure Fund (EAIF), or the Climate Investment Fund (see, for example, Globeleq 2023; IFC 2021; Scatec 2018). On its own, EDM has relied largely on donations and concessional and semi-concessional financing from a range of international entities, from BADEA (the Arab Bank for Economic Development in Africa) to the Export Import Bank of India, the African Development Bank, the World Bank, the EU, and several European countries (EDM 2020). Overall, the diversity of partners, funding sources, and types of financing instruments illustrates the substantive effort put into the electrification of Mozambique through what is essentially a handful of projects. After all, Mozambique needs ten times that amount over the next 25 years to complete its pipeline of electricity projects and meet its energy needs.

The paper proceeds to examine how the national utility EDM and the GoM have engaged in such reforms, first in the on-grid sector and then the off-grid sector. It will show how these actors engaged with the dominant financial value regime over the last 3 decades, sometimes willingly, other times reluctantly, opportunistically, or only pragmatically. Along the way, we will point out how this strategic engagement with the dominant financial value regime seemingly reinforced the heterogeneity of country’s energy landscape and exacerbated energy injustice.

### Aligning the on-grid sector

The GoM has been very pragmatic and even opportunistic about the way it aligns the on-grid sector with the dominant financial value regime promoted by the likes of the World Bank and the donor community. We examine three key processes: sector regulatory reforms; EDM creditworthiness; and EDM project pipeline and bankability.

Electricity sector regulatory reforms have been ongoing since the 1990s and have sought to align the demands of the Wall Street Consensus with the GoM’s preferred economic accumulation model. The first post-1990 reform, the 1997 Electricity Law, introduced the principles of sector liberalization, privatization, and vertical disintegration (República de Moçambique 1997). However, private participation never really took off until the approval of legislation to facilitate PPPs for large projects and business concessions in the early 2010s (República de Moçambique 2011, 2012, 2013). This opened the way for IPPs to generate electricity and sell it to the national utility EDM, usually at a high profit. Under the pretense of spreading the benefits of PPPs across Mozambican society, this legislation required a participation of Mozambican capital in each PPP for large projects (República de Moçambique 2012, Articles 64.º e 65.º). Government-related business elites and their supporters began partnering in IPP consortia, leading to their personal enrichment (Nhamire and Mosca 2014). One example here is the private company Gigawatt Moçambique SA, owner of an energy park in southern Mozambique, whose initial shareholders included a South African energy company, the company of a former energy minister, and the company owned by the family of the then President of the Republic (Cortês 2018). Scholars have argued that the politics of selective access to business opportunities by government-aligned actors has made the domestic private sector dependent on the state for survival and vice-versa (Cortês 2018; Pitcher 2012), which has undercut broader developmentalist agendas, namely in terms of affordable access to electricity to facilitate poverty alleviation. This finding seems to align with the view noted earlier that global South state actors actively seek to contest or subvert efforts as international financial subordination by more powerful actors.

An example of this tension is EDM’s difficulty in meeting the government’s competing demands, while maintaining its financial viability. EDM’s 10-year strategy for 2018–2028 identified three government-mandated goals (see EDM 2018): to operate as a commercially viable and competitive utility; to achieve SDG7; and to support the government’s aim of becoming an energy export hub for southern Africa. In practice, meeting these goals is a challenge. For one, EDM faces a chronic problem of liquidity, a well-known issue and cause of concern not just to its leadership, but also to donors and investors (The World Bank 2015). One reason it struggles to keep its books balanced is that its tariffs are regulated by the government, which has kept them below cost-recovery figures for fears of public backlash and destabilization of its increasingly authoritarian regime (see Brito et al. 2014). Indeed, in 2018, EDM charged its customers an average of USD 0.102/kWh through the government regulated tariff, about 80% of its average operation cost of USD 0.128/kWh (EDM 2019). The revenue imbalance was further exacerbated by the fact that EDM generated only 9% of its own supply and acquired 38% from IPPs, which charged an average unit price of USD 0.180/kWh (EDM 2019). Concurrently, that same year, EDM exported 1.5 times the amount it was generating for internal supply to the Southern Africa region (EDM 2019). In other words, EDM exported a significant amount of electricity that it then had to acquire from IPPs at an exorbitant price. With the government resisting a full update to electricity tariffs on par with cost-recovery principles, EDM has struggled to meet its existing liabilities and to demonstrate its creditworthiness.

**Fig. 1:**
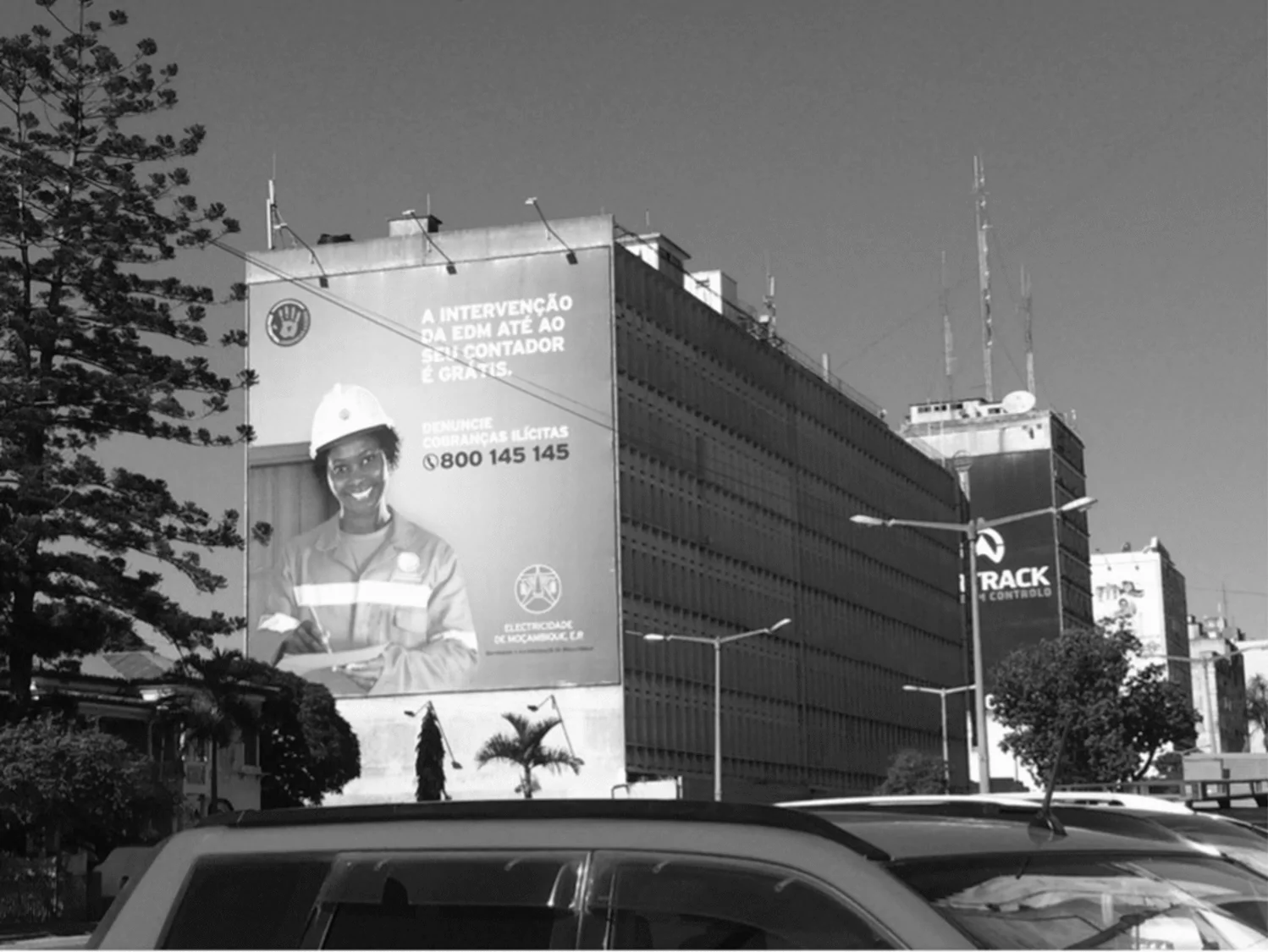
EDM anti-corruption outdoor publicity on the side of its Maputo headquarters. It states: “EDM’s intervention up to your meter is free. Report illegal charges. Call 800 145 145.” Photo © by Idalina Baptista

**Fig. 2:**
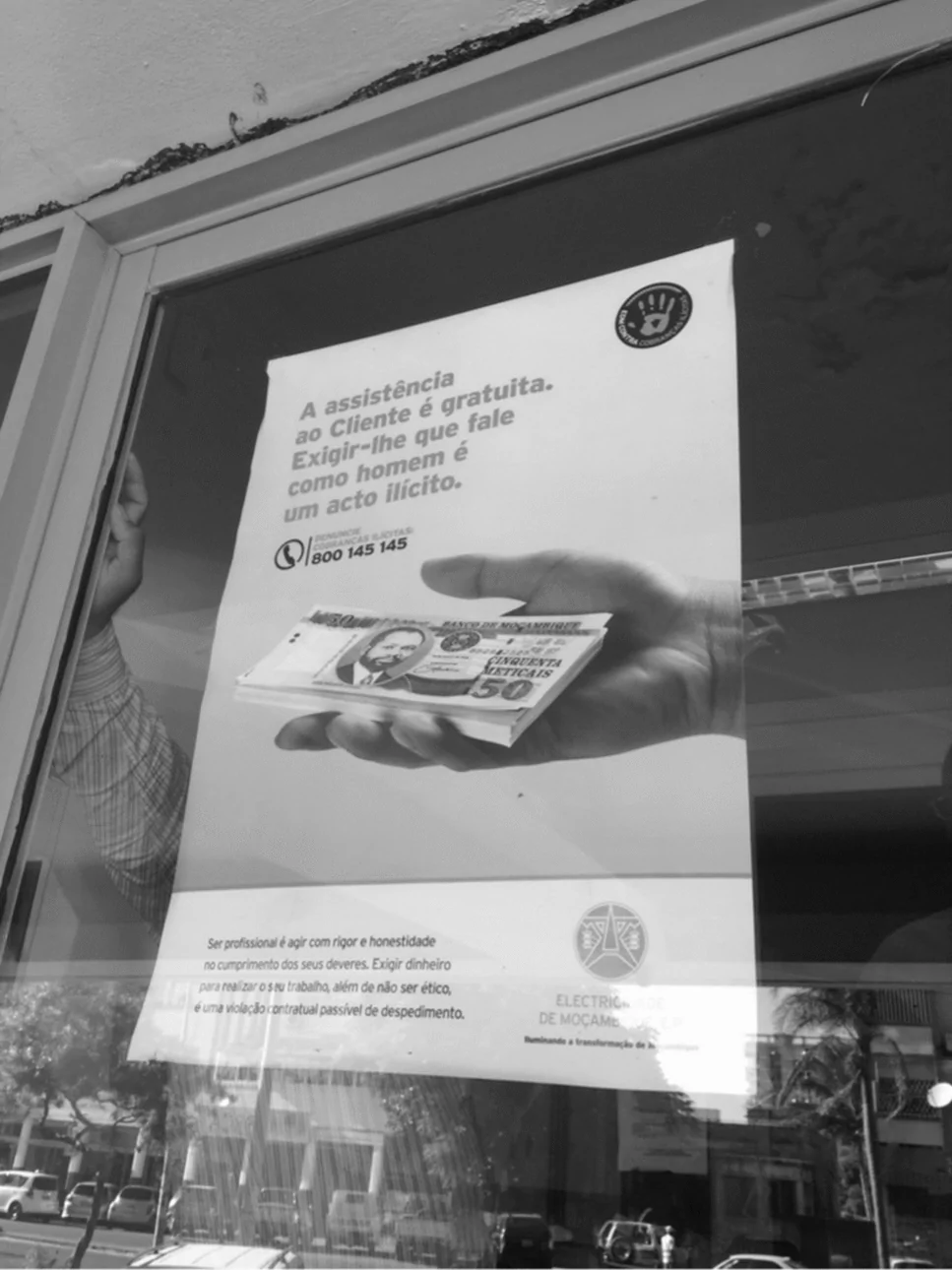
EDM anti-corruption poster on the Maputo headquarters’ customer shop. It states: at the top, “Customer assistance is free. Requiring customers to speak like a man is an illicit act. Report illegal charges. Call 800 145 145;” at the bottom, “To be professional is to act with rigor and honesty in the fulfillment of one’s duties. Demanding money to carry out one’s job is not only unethical but also a contractual breach subject to dismissal.” Photo © by Idalina Baptista

In this context, EDM has had to find alternative ways to create the conditions to raise finance for its extensive portfolio of infrastructure projects. One way has been to engage in organizational restructuring. Viegas Filipe et al. (2021) have shown how EDM adapted the organizational structure of the company to isolate the loss-making part of the business from the commercially viable one. This was introduced alongside measures to bring in greater transparency, meritocracy, and integrity to EDM’s operation—all principles neatly aligned with the dominant financial regime. These included: having renowned accounting and auditing firms (e.g., KPMG and Ernst and Young) review its annual accounts and financial reports and making these public on the company’s website; launching international, merit-based recruitment processes to curb accusations of political influence peddling; and approving a Code of Ethics for its staff. The latter appealed to workers’ moral values, admonishing them to act ethically and according to their work contract or to be subject to disciplinary procedures. The Code was followed up by a publicity campaign urging customers to resist corrupt behavior by EDM staff. For example, it urged customers not to engage with bribe requests, even if EDM workers challenged customers’ masculinity to exact monetary compensation in exchange for preferential treatment (Figs. 1 and 2).

**Fig. 3:**
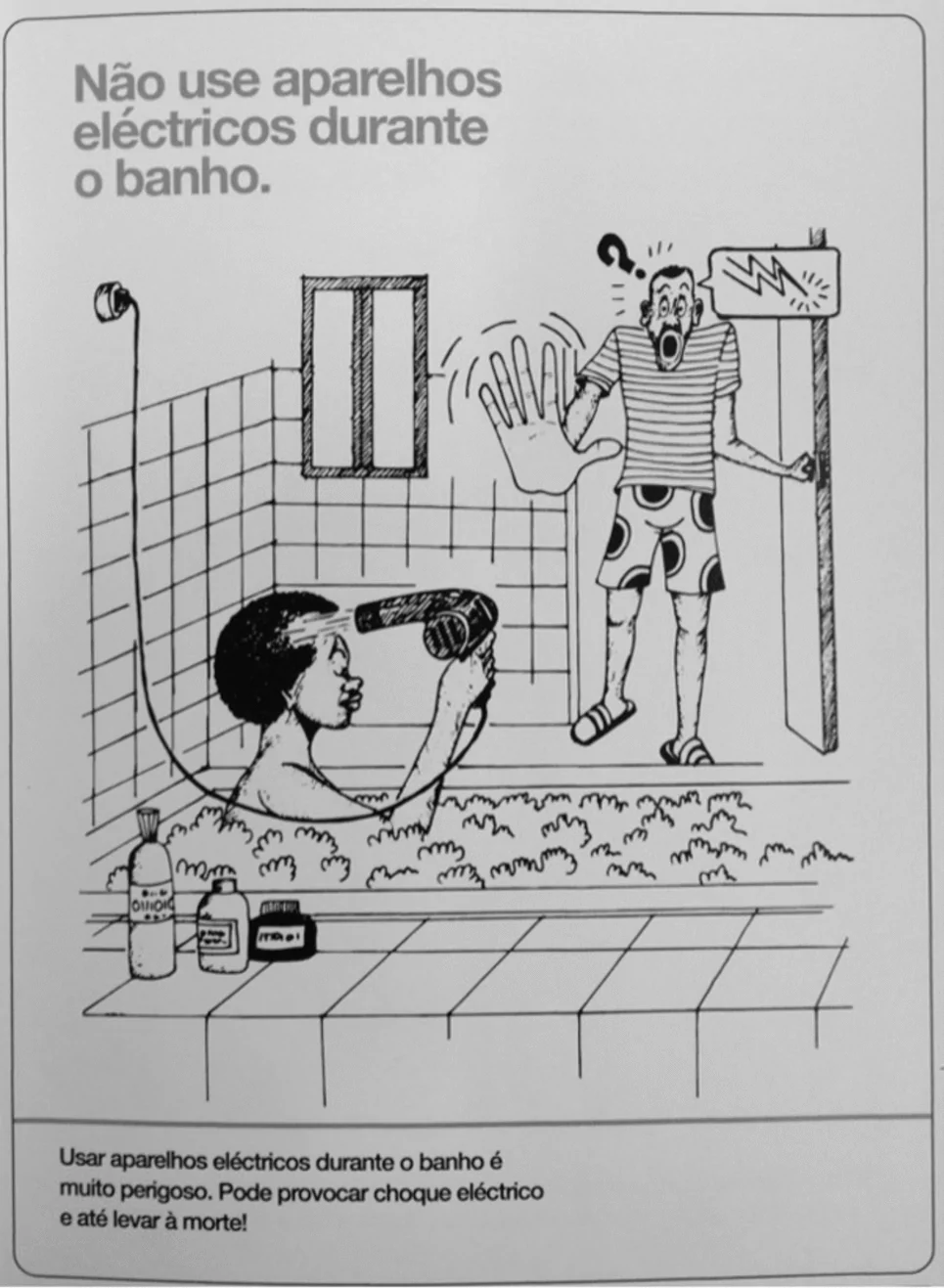
“Don’t use electric appliances while bathing. Using electric appliances while bathing is very dangerous. It can cause [an] electric shock and even lead to death!”. Source: EDM (2017)

**Fig. 4:**
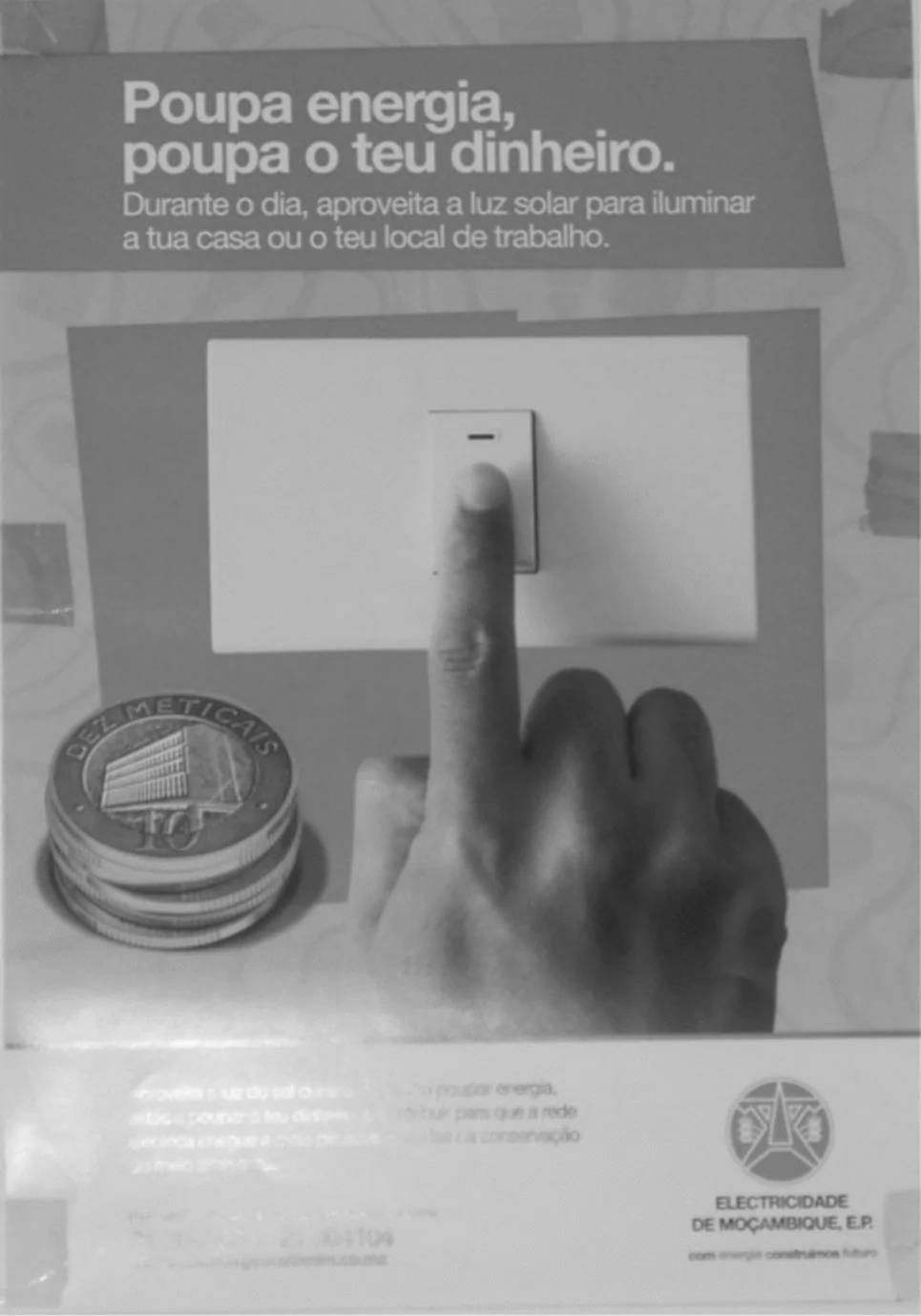
“Save electricity, save your money. During the day, take advantage of daylight to light your house or office”. Photo © by Idalina Baptista

Concurrently, customers were urged to adopt certain behaviors in their electricity consumption practices, a key aspect for the metrification (and commensurability) of electricity supply and revenue. Two examples are EDM (2017)’s *Citizen’s Manual for the Prevention of Electric Accidents* (Fig. 3) and the electricity savings campaign (Fig. 4). While the Manual embodies a broader concern with making customers electricity-literate, the electricity savings campaign seeks to reduce ‘wasteful’ electricity consumption in the face of limited supply. This is a rather ironic anxiety in the context of a population that has a very low consumption per capita. EDM has pursued these aims alongside efforts to increase revenue collection and curb electricity theft and illegal connections, namely through the introduction of prepaid metering (Baptista 2015, 2019). While prepayment has had some relative impact in stabilizing revenue collection among the general population, EDM continues to struggle with collecting payment from state institutions, such as the ministries of Health and Defense (Salite et al. 2021).

Overall, the diversity of directions of intervention reveals EDM’s understanding of the complex challenges of aligning itself with the dominant financial value regime. It was not just a matter of appearing to be transparent, accountable, and competitive. Its internal structures, from the top directorship positions to the field-level worker, and its own customer base all had to be aligned morally, ethically, and normatively with the dominant financial value regime. However, not all customers were treated the same, leading to the emergence of a morally heterogeneous energy landscape: whereas state institutions resist payment, lower income citizens are cut-off from supply for illegal connections or non-payment.

Despite these efforts toward meeting normative aspects of creditworthiness, project bankability remained a key issue for EDM. As noted earlier, the Integrated Master Plan’s estimated investment of USD 34 billion covers all levels of the on-grid system as well as improvements to EDM operations (República de Moçambique 2018a). The Plan lists several generation and transmission projects, many of which have been in Mozambique’s wish list since independence (and some even before). Finding willing finance capital thus required the wish-list of projects to meet favorable assessments of their risk profile or financial viability by potential investors. To this end, EDM enlisted the support of the Japanese International Cooperation Agency to develop a technical study detailing all aspects of its operation, supply and demand dynamics, and preliminary project profiles and finance-related issues (e.g., rate of return on assets; currency and exchange rate; interest rates and debt financing; projected power sales, tariffs, and losses; consumer price indexes; depreciation; etc.). The technical detail involved in this study, even when not detailed enough for individual projects, signaled an understanding of how project bankability *ought* to be represented. The Plan is thus seeking to speak the same language as that of the financial value regime it must align itself with.

In line with the requirements for project bankability, EDM pushed the GoM to develop several legislative efforts to improve the transparency and reliability of the electricity sector’s regulatory framework. These efforts began bearing fruit when the government approved the creation of a new energy regulator, ARENE, in 2017 (República de Moçambique 2017), followed by the approval of the National Electrification Strategy 2018–2030 (República de Moçambique 2018b). Further, the new 2022 Electricity Law clarified the division of labor between on-grid and off-grid electrification to be led, respectively, by EDM and by FUNAE, the National Energy Fund (República de Moçambique 2022). EDM would be responsible for connecting new customers located within 100 m of the existing grid with its own resources. The company would request subsidies from the government or donors to expand the grid to customers outside those 100 m. FUNAE was expected to lead off-grid electrification and then transfer mini-grids to EDM (see next section). These legislative efforts seem to show EDM’s own attempts at retaining some agency considering the demands of the GoM and of the dominant financial value regime.

In practice, this division of labor meant two things. On the one hand, the new legislation enshrined the division between on-grid profit-making and off-grid social/loss-making projects which EDM had initiated with its own organizational restructuring a few years earlier. On the other hand, in seeking to sustain EDM’s creditworthiness and the bankability of electrification projects, the legislation also furthers the fragmentation of electricity access across space. Since electrification beyond 100 m of the electricity grid depends on access to government subsidies, there is limited guarantee it will proceed at all. A similar problem seems to be emerging with off-grid electrification, to which we turn next.

### Aligning the off-grid sector

If it has been hard to raise private finance for on-grid projects in local and global markets, it is even harder to come by finance for off-grid projects. Until recently, FUNAE-led off-grid electrification had a limited impact on rural communities, which had an electrification rate of only 5% (República de Moçambique 2023). The donor community, currently a substantive, if modest, supplier of capital for new off-grid renewables projects, is trying to address finance bottlenecks, but progress is slow and seems to fall well short of demand. Current estimates put donor contribution for finance of off-grid renewables in the region of EUR 212.5 million (ALER and AMER 2023), dispersed over some 24 donor programs that do not seem to meet the institutional realities of Mozambique’s electricity sector (see Gebreslassie et al. 2022). For example, in 2023, the German development bank KfW and the Global Environment Facility’s (GEF) Environmental Fund, in partnership with UNIDO and FUNAE, financed two credit lines dedicated to renewable energy projects through one of Mozambique’s commercial banks. However, because demand for finance was high, both credit lines quickly exhausted all their financing capacity (ALER and AMER 2023). Another example is the EU-funded *GET.invest* program, which provides hand-holding support, model business cases and toolkits to help domestic energy operators attract financing (GIZ 2023).

Given the limited impact of these efforts, the donor community has been pushing the GoM to clarify the regulatory environment in the hopes this would attract private investors. As a consequence, new legislation was passed in 2021 to define the regulatory environment for the off-grid sector (República de Moçambique 2021), followed by the GoM’s 2023 Off-grid Electrification Plan (República de Moçambique 2023). The new legislation sought to open a pathway for private and donor-incentivized energy operators to supply green mini-grids up to 10 MW and other autonomous energy services, including Solar Home Systems (SHSs). According to the Off-grid Electrification Plan, universal access to electricity by 2030 would be realized by connecting 68% of the population to the grid (via EDM), with the remaining 32% accessing electricity via off-grid options (via FUNAE), mostly through renewables—19% via SHSs and 13% via mini-grids (República de Moçambique 2023). This division retains a similar spatially differentiated access as the 2018 National Electrification Strategy for the on-grid sector: potential consumers located within 30 km from the national grid may be connected to it, whereas those located beyond that distance will be connected via mini-grids; however, in cases of low population density, where distance between households is over 350 m, households will access electricity via a SHS (República de Moçambique 2023).

Obviously, every plan is aspirational and indicates not a future reality but a direction of travel. In this case, reading the 2018 National Electrification Strategy alongside the 2023 Off-grid Electrification Plan, it is fair to conclude that there will be a government-sanctioned spatially differentiated access to electricity: the farther away one is to the existing grid, the greater the chance that access will be fulfilled through an off-grid or individual home solution. In principle, there is nothing normatively negative in accessing electricity via off-grid or individual home solutions instead of accessing it through the grid, so long quality and affordability of service allow people to flourish and achieve the wellbeing they desire (Castán Broto et al. 2018). The new legislation offers a patchy, fuzzy pathway for the electrification of distant, rural communities, whose access to electricity may just be delivered via off-grid projects. In practice, this may mean that access by these communities may not materialize or materialize in unaffordable, unreliable conditions. In other words, what the GoM seems to be aiming at with this new legislation is to channel investment to the on-grid system via EDM and IPPs, through which larger profits can be made, and leaving a substantive part of the population, the least profitable segments, to be delivered by others—i.e., FUNAE, the donor community, and smaller renewable energy operators. Unlike the pragmatic and potentially pragmatic forms of service differentiation reported by Jaglin (2008) for Cape Town, this spatially differentiated access foretells an increasingly heterogeneous energy landscape with uneven levels of service quality and affordability in future.

This government-sanctioned spatially differentiated access to off-grid electrification seems to reflect the GoM’s disinterest in the sector as means of economic accumulation. Indeed, it is notable that the 2021 off-grid regulatory framework has no requirements for investments to be participated by Mozambican capital as it was the case of the 2010s legislation for the large-scale projects involving PPPs. Moreover, it has taken some time for the GoM to approve additional regulations and tax incentives outlined in the 2021 off-grid regulatory framework. This disinterest and uncertainty are seen with concern by off-grid stakeholders, for whom the new legislation is a good start, but not a sufficient condition for investment. In interviews conducted with off-grid stakeholders, we identified two main sticking points. On the one hand, stakeholders highlighted that favorable financing conditions had to meet the reality of the local context. They believe these conditions must be in place because off-grid operators—like utilities and governments—must also meet the requirements of creditworthiness and project bankability. On the other hand, stakeholders had yet to understand how the GoM was going to exercise the power over the purse with regards to tax benefits for the off-grid sector.

With regards to promoting favorable financing conditions, off-grid operators are also heavily reliant on donor projects and financing to subsidize costs and establish themselves in the market whether this be through the supply of grants, debt or equity loans, guarantees, or technical assistance. As with on-grid electricity supply, bankability permeates new off-grid development efforts. For instance, obtaining financing from commercial banks is prohibitive for most businesses due to high interest rates and perceived high risks of their long-term viability. Moreover, the prevailing taxation regime directly impacts the affordability of off-grid electrification. As interviewees explained, if capital expenditure costs and taxes are to be recovered, they must be passed on to Mozambican consumers, the majority of whom have very low or limited income (Howe et al. 2024). Some of the interviewees feared that off-grid solutions, especially mini-grids, may end up connecting the segments of the population that have a higher ability to pay for the investment. Alternatively, interviewees suggested, investors would have to secure a substantive off-taker (e.g., a large consumer such as a local industry) capable of anchoring the investment in a consistent stream of demand, while also providing to lower income consumers.

The second sticking point relates to the taxation framework and the subsequent bankability of off-grid projects. Off-grid actors seemingly keen to invest in Mozambique face significant uncertainty regarding fiscal arrangements (e.g., taxation, custom duties) and technical arrangements (e.g., specifications for future interconnection with the grid), all of which hamper their ability to conduct sound project feasibility studies. While the off-grid legislation was approved in 2021, it took the GoM many months, until June 2023, to approve all 27 regulatory specifications integral to the operationalization of off-grid projects (Howe et al. 2024). To address the taxation shortcomings, donors have been lobbying the GoM to reduce import duties and VAT to support the off-grid sector. The GoM has made some ambiguous gestures to this end. In August 2022, it announced an economic stimulus package offering a VAT exemption on electrical imports specifically targeted at off-grid energy investments (Ministério da Economia e Finanças 2022). Yet, there have been lengthy delays in the operationalization of this measure, meaning that off-grid energy operators are left in limbo on how best to take advantage of these fiscal incentives. As with EDM’s approach to become creditworthy, the GoM has shown itself as being pragmatic in aligning itself with the value regimes of donor finance. The GoM seems to be playing a careful balancing act between responding to the demands of the financial value regime promoted by donors but without committing too much for the budding off-grid energy sector.

This reluctant approach by the GoM may be best understood in the broader context of the political economy of Mozambique’s energy sector and its own preferred strategy of economic accumulation. As noted above, the GoM has favored public–private collaborations that can facilitate a regime of resource extraction and has focused much less on enabling renewable energy transitions. However, this calculation may be shifting, if only for opportunistic reasons. In the broader energy landscape, Mozambique is expected to become the third-largest producer of natural gas in Sub-Saharan Africa by the mid-2030s (Deloitte and Touche 2023) alongside the global push to transition to net zero/decarbonized economies in support of the Paris Climate Agreement. Against this backdrop, the GoM launched an ambitious energy transition strategy in December 2023, seeking more than USD 80 billion in public and private investment by 2050 (AfDB 2023). The strategy aims to expand renewable energy capacity through hydro, solar and wind power plants to offset the share of fossil fuels (coal and gas) in the energy matrix. For the GoM, the development of the gas reserves is a non-negotiable, especially when part of the country’s current debt finance has been tied to future fossil fuel revenue (Foster et al. 2024). Therefore, the GoM will have to negotiate the rapidly emerging asset class of renewable energy infrastructures without allowing the financial value regimes of donors and investors impinge on its own electrification agenda.

In sum, the various interventions of the GoM in the off-grid sector seem to signal a willingness to align the electricity sector with the dominant financial value regime, albeit in its own terms. The GoM’s legislative efforts to date have contributed to lay only the most basic foundations for the regulatory environment to be recognized as adequate by potential financiers, thus circumventing as much as possible the dominance exercised by the Wall Street Consensus. Some scholars argue that creating trust regarding the effectiveness of the regulatory framework—and, by extension, trust in government institutions and their intentions—is crucial if investors are not to be tempted to shift their investments elsewhere (Shenga et al. 2024). Yet off-grid operators face their own constraints in meeting the requirements of creditworthiness and project bankability. Any delays (perceived or real) in implementing off-grid electrification policies, such as the tax incentives, the economic stimulus package, or the national energy transition strategy, could lead to a slowdown in off-grid investment and electrification.

## Conclusion

Financing has been part of Africa’s electricity infrastructure development since the nineteenth century, so it is unsurprising it remains key to the continent’s drive for universal electrification. However, since the 1980s, we have been witnessing the entrenchment of a financial value regime that treats electricity infrastructures mainly as assets not as public goods with a social purpose (Ashton et al. 2012; Baptista 2024). Therefore, while the use of financ*ing* in infrastructure development is not inherently problematic, the current financ*ialization* of infrastructures has substantive implications for the prospects of universal electrification and for energy justice, which are central to meeting sustainability agendas (Sahle et al. 2024).

Drawing on the case of Mozambique, this article examined the strategies deployed by state actors to align themselves with the dominant financial value regime and the implications this has for the country’s energy landscape, particularly in terms of environmental sustainability and social justice. The article reviewed three main process of alignment that spanned across the on-grid and off-grid sectors: regulatory reforms to ‘de-risk’ the political environment and create electricity markets; organizational changes to improve the creditworthiness of financing recipients (i.e., on-grid utilities and off-grid operators); and strategies and instruments to enhance project bankability. The analysis of the Mozambican case suggests that in the process of aligning with the dominant financial value regime, local actors use pragmatic, opportunistic, or ambiguous strategies that place them in the best position to attract financing and/or to maximize profits, while limiting the dominance of the so-called Wall Street Consensus. One significant impact of these strategies in the Mozambican context was the government-sanctioned codification into law of the country’s uneven and unequal heterogeneous energy landscape. As it currently stands, the GoM is placing its electrification efforts on profitable ventures, while relegating the electrification of hard to reach rural and/or lower income populations to less profitable operations that may never come to fruition. In the same vein, given the GoM’s interest in expanding electrification through gas projects, efforts to facilitate the emergence of an off-grid renewable electricity sector may be no more than window dressing. Therefore, while there may be merits in offering differentiated electricity services to different segments of the population, the current set-up seems to suggest the GoM is uninterested in maximizing the social justice potential of the legislative changes it approved.

While the specific findings of the Mozambique case cannot be generalized, they offer three key insights into the trade-offs and negotiations that mediate Africa’s pathways to universal electrification. First, reforms to facilitate private involvement in Africa’s electricity sector have been ongoing for some three decades now. Yet, development assistance has been the norm and private financing the exception. It is unclear whether private financing—whether by foreign or national investors—can be persuaded to invest in the least profitable market segments—that is, to service the 66% of Africans who currently lack electricity access. We may need to come to terms with the fact that, under the current financial value regime, the necessary private financing may not be forthcoming. Second, this insight reveals similar concerns to those already raised by scholars looking at climate finance more broadly (e.g., Knuth and Taylor 2023), especially in what concerns the socio-spatial effects of financial flows. In this vein, our case study suggests that alignment with the dominant financial value regime may also impact the spatial distribution of electricity infrastructures and/or the diversity of service provision afforded to different people. In the case of Mozambique, we see the consolidation of existing inequalities evident in the GoM’s accumulation model. We have modest hopes this could be otherwise elsewhere. Indeed, and finally, the emergence of reliable and affordable off-grid renewable energy solutions may drive state actors to accept and deploy a diversity of configurations of electricity service provision, but for the wrong reasons. A heterogeneous energy landscape may provide the flexibility needed to deal with the climate crisis and meet the energy needs of communities at the local level. Yet, it can also be used strategically to leave behind those most in need. 
